# Circulating calcitonin and carcinoembryonic antigen m-RNA detected by RT-PCR as tumour markers in medullary thyroid carcinoma

**DOI:** 10.1054/bjoc.2001.2111

**Published:** 2001-11

**Authors:** J Bojunga, C Dragan, P M Schumm-Draeger, K H Usadel, K Kusterer

**Affiliations:** Department of Medicine I, Goethe-University, Theodor-Stern-Kai 7, Frankfurt, 60590, Germany

**Keywords:** polymerase chain reaction, reverse transcription, medullary thyroid carcinoma, molecular diagnostics

## Abstract

Detection of local relapse or metastasis in medullary thyroid carcinoma (MTC) continue to pose a major diagnostic challenge. Besides established diagnostic studies such as serum calcitonin (CT) and carcinoembryonic antigen (CEA), molecular detection of circulating tumour cells may be an additional diagnostic tool for the early detection of disease recurrence. We performed reverse transcription-polymerase chain reaction (RT-PCR) on blood samples from patients diagnosed with MTC disease using primers specific for CT and CEA, respectively. CT mRNA was not detectable in peripheral blood of all patients with MTC (*n* = 11) and all controls (*n* = 32). CEA mRNA was significantly more often detected patients with MTC (72.7%) than in controls (34.4%; *p* = 0.038; Fisher exact test). With an example of a patient with MTC and massive tumour mass in the neck we demonstrate the failure of detection of CT mRNA over a period of 6 months, whereas CEA mRNA could be detected in peripheral blood of this patient. As a consequence, CT mRNA detected by RT-PCR in the peripheral blood can not be recommended as a tumour marker in MTC. However, the use of carcinoembryonic mRNA may provide a significant improvement in diagnosis of recurrent disease in MTC. © 2001 Cancer Research Campaign   http://www.bjcancer.com


*British Journal of Cancer* (2001) **85**(10), 1546–1550
© 2001 Cancer Research Campaign
doi: 10.1054/ bjoc.2001.2111, available online at http://www.idealibrary.com on IDEAL

### Circulating calcitonin and carcinoembryonic antigen m-RNA detected by RT-PCR as tumour markers in medullary thyroid carcinoma

**J Bojunga, C Dragan, PM Schumm-Draeger, KH Usadel and K Kusterer**

Department of Medicine I, Goethe-University, Theodor-Stern-Kai 7, 60590 Frankfurt, Germany

**Summary** Detection of local relapse or metastasis in medullary thyroid carcinoma (MTC) continue to pose a major diagnostic challenge. Besides established diagnostic studies such as serum calcitonin (CT) and carcinoembryonic antigen (CEA), molecular detection of circulating tumour cells may be an additional diagnostic tool for the early detection of disease recurrence. We performed reverse transcription-polymerase chain reaction (RT-PCR) on blood samples from patients diagnosed with MTC disease using primers specific for CT and CEA, respectively. CT mRNA was not detectable in peripheral blood of all patients with MTC ($n$ = 11) and all controls ($n$ = 32). CEA mRNA was significantly more often detected patients with MTC (72.7%) than in controls (34.4%; $p$ = 0.038; Fisher exact test). With an example of a patient with MTC and massive tumour mass in the neck we demonstrate the failure of detection of CT mRNA over a period of 6 months, whereas CEA mRNA could be detected in peripheral blood of this patient. As a consequence, CT mRNA detected by RT-PCR in the peripheral blood can not be recommended as a tumour marker in MTC. However, the use of carcinoembryonic mRNA may provide a significant improvement in diagnosis of recurrent disease in MTC. © 2001 Cancer Research Campaign http://www.bjcancer.com

**Keywords**: polymerase chain reaction; reverse transcription; medullary thyroid carcinoma; molecular diagnostics

Medullary thyroid carcinoma (MTC) is a rare disease and accounts for 3–10% of all thyroid carcinomas (Hundahl et al, 1998). Because MTC can only be cured surgically and no effective radio- or chemotherapy are available, mortality of MTC is higher than of differentiated thyroid carcinoma and accounts for up to 13.4% of all deaths related to thyroid malignancies (Marsh et al, 1995). Serum level of basel as well as of pentagastrin-stimulated calcitonin is as established tumour marker in MTC, which is supplemented by estimation of serum levels of carcinoembryonic antigen (CEA). Hereby, early detection of metastases and local disease relapse remain to pose a major clinical problem in MTC. Searching for local relapse of distant metastases is often very difficult and includes laboratory tests as well as selective venous catheterization, ultrasound, octreotide scan, computer tomography and magnetic resonance imgaging. Therefore, molecular detection of circulating tumour cells may represent a new approach in order to improve early diagnosis of metastasized MTC.

In a recent study (Bojunga et al, 2000) we could show that detection of thyroglobulin mRNA in peripheral blood of patients with differentiated thyroid carcinoma by RT-PCR correlated with the diagnosis of metastasized thyroid carcinoma, although high assay senitivities resulted in non-specific transcript-amplification. In this study we now tested whether detection of circulating calcitonin and carcinoembryonic mRNA in peripheral blood of patients with medullary thyroid carcinoma may be a diagnostic tool for identifying patients with local disease recurrence or metastasized MTC.

*Received 8 May 2001*
*Revised 23 July 2001*
*Accepted 7 August 2001*

*Correspondence to:* J Bojunga

### METHODS

### Cell culture

TT cells, a medullary carcinoma cell line producing high levels of calcitonin and carcinoembryonic antigen, was purchased from the American Type Culture Collection. Cells were cultured in RPMI 1640 medium supplemented with 15% fetal calf serum, 10 mM HEPES, 6 mM glutamine. The medium was changed every 3 days and cells were harvested at confluence.

### Patients

After approval by the human study committee 11 patients with medullary thyroid carcinoma and 32 control subjects without evidence or history of thyroid disease were studied. Diagnosis of cancer was based on histological examination of the surgical specimen. Diagnosis of healthy individuals was based on an appropriate ultrasound examination and normal serum markers (calcitonin, carcinoembryonic antigen, thyroid-stimulating hormone (TSH), total thyroxin (T4) and triiodothyronine (T3)). All patients with a diagnosis of medullary thyroid carcinoma had been treated surgically and were followed in the Department of Endocrinology at the Johann Wolfgang Goethe-University Hospital and volunteered to participate in the study.

### Blood samples and RNA extraction

The interval between surgery and collecting of blood samples was >1 month in all patients with MTC. Blood samples were prepared as described earlier (Bojunga et al, 2000). In brief, approximately 9 ml venous blood was obtained from each patient in the morning prior to any diagnostic procedure in our outpatient clinic at a routine appointment. Blood samples were immediately mixed with EDTA to prevent coagulation and cooled down to 4˚C. Nucleated cells were isolated using a Percoll gradient (Pharmacia, Uppsala, Sweden) at a density of 1.09 g/ml within 2 h after withdrawal of blood. Serum for calcitonin, CEA and thyroid hormone measurements was obtained using the supernatant after centrifugation. Total cellular RNA was extracted using RNeasy blood kit (Qiagen, Hilden, Germany) according to the suggestions of the manufacturer and RNA was resuspended in 50 µl RNase-free water. Optical transmission of the final RNA-preparation was determined three times at 260 nm using a Gene-Quant calculator (Pharmacia Biotech, Freiburg, Germany) and the RNA concentration was calculated.

### Oligonucleotide primers and probes

Calcitonin primers were used as described elsewhere (Bracq et al, 1993). CEA primers were custom designed according to the human CEA cDNA sequence reported by Barnett et al (Gene Bank accession number M29540). All primers were synthesized and purified by MWG-Biotech (Ebersberg, Germany). CT primers bind exon 3 and 4 respectively, generate a 291 bp fragment and span a genomic DNA sequence so that cDNA and contaminating genomic DNA amplification products can be distinguished by size. CEA primers bind exon 2 and 4 respectively and generate a 328 bp fragment. To assess the intactness of amplifiable RNA two intron-spanning glyceraldehyde-3-phosphate-dehydrogenase (GAPDH) primers that generate a 300 bp fragment (Gene Bank accession number M33197) were used. The specificity of the primer sets for CT, CEA and glyceraldehyde-3-phosphate-dehydrogenase, respectively, was verified by basic local alignment search tool (BLAST) computer search (Altschul et al, 1990). The sequences of these PCR primers are listed in Table 1.

### Reverse transcriptase–polymerase chain reaction

Approximately 1.5 µg of total RNA was reverse transcribed in a final volume of 20 µl containing 4 µl 5× buffer (250 mM Tris-HCl pH 7.5, 375 mM KCl, 15 mM $MgCl_2$, 50 mM DTT), 20 units recombinant RNase inhibitor (Promega, Madison, WI, USA), 0.5 mM dNTP, 2.5 µM oligo dT and 200 units RNAse H minus M-MLV reverse transcriptase (Promega). The following PCR was performed in a total volume of 50 µl containing 3 µl of the RT-solution, 5 µl 10× reaction buffer (200mM Tris-HCl pH 8.4, 500 mM KCl), 0.5 µM forward and reverse primer, 0.2 µM of each dNTP, 2.5 units Taq-DNA polymerase and 1.5 mM $MgCl_2$ (Life Technology, Paisley, Scotland) using a tube-controlled DNA thermal cycler (AGS-Hybaid, Heidelberg, Germany). For amplification of CEA cDNA, samples were subjected to 3 min of denaturation at 94˚C followed by 30 cycles of 45 s at 94˚C, 30 s at 55˚C and 1 min 30 s at 72˚C, followed by additional 10 min at 72˚C. For amplification of CT cDNA, samples were subjected to 3 min of denaturation at 94˚C followed by 10 cycles of 45 s at 94˚C, 30 sec at 65˚C decrescendo cycle-by-cycle to 55˚C and 1 min at 72˚C, followed by 20 cycles of 45 s at 94˚C, 30 s at 55˚C and 1 min at 72˚C followed by additional 10 min at 72˚C. In each experiment water was used as a negative control.

### Analysis of amplified CT/CEA cDNA

10 µl PCR product was mixed with 2 µl 6× loading dye (MBI Fermentas, Vilnius, Lithuania) and run for 60 min on an 1.5% agarose gel in TBE buffer (0.1 M Tris pH 8.4, 90 µM boric acid, 1 mM EDTA). The gel was stained with ethidium bromide and the bands corresponding to the amplified fragments were visualized on UV-table. Sequencing of amplified CT/CEA fragments was performed using a commercial sequencing service (SeqLab, Göttingen, Germany).

### Calcitonin/carcinoembryonic antigen RT-PCR assay sensitivity

RT-PCR assay sensitivity was determined by mixing total RNA isolated from whole blood of a negative control patient (negative with CT and CEA RT-PCR) with total RNA isolated from TT cell line in serial dilutions of 1:2, 1:5 and 1:10 steps, respectively. RT-PCR was performed and analysed as described above. Assuming that one TT cell contains approximately 10 pg total RNA, the lower detection limit of the described RT-PCR assay was calculated as TT cells/ ml whole blood.

### Statistics

Statistical comparisons were done using Sigma-Stat statistical software (Jandel Scientific, Erkrath, Germany). Contingency tables were used to assess the association between RT-PCR positivity rates and research subject category. Fisher exact test was used to compare the distributions in contingency tables that had 5 or less expected observations in one or more cells. $P < 0.05$ was considered significant.

### RESULTS

### Detection of CT and CEA transcripts by RT-PCR

After optimization of conditions for reverse transcription and polymerase chain reaction we were able to detect calcitonin as well as

**Table 1** Oligonucleotides and primers

| Oligonucleotide | Sequence | Size of amplicon | Gene Bank accession |
|---|---|---|---|
| Calcitonin primer (+) | 5′ GGCAGCCTCCATGCAGCACC 3′ | 291 | J00109 |
| Calcitonin primer (–) | 5′ CCAGGTGCTCCAACCCC 3′ | | |
| CEA primer (+) | 5′ GACTCAGGACGCAACCTACC 3′ | 328 | M29540 |
| CEA primer (–) | 5′ ATTGCTGGAAAGTCCCATTG 3′ | | |
| GAPDH primer (+) | 5′ CGTCTTCACCACCATGGAGA 3′ | 300 | M33197 |
| GAPDH primer (–) | 5′ CGGCCATCACGCCACAGTTT 3′ | | |

© 2001 Cancer Research Campaign

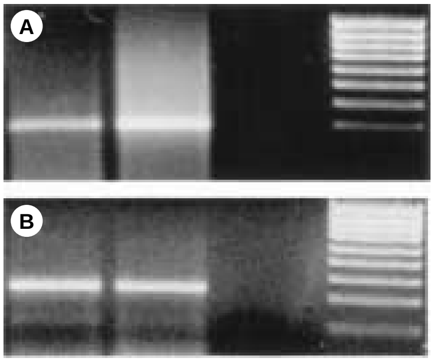

**Figure 1** Agarose gel electrophoresis of CT (**A**) and CEA (**B**) reverse-transcriptase polymerase chain reaction products (291 bp and 328 bp, respectively) derived from total RNA from TT-cell line carcinoembryonic antigen transcripts as the expected 291/328 bp amplicons in total RNA extracted from TT-cell culture by agarose gel electrophoresis (Figure 1). PCR products were sequenced and their identity was consistent with human calcitonin/carcinoembryonic antigen cDNA sequence (Gene bank accession numbers see Table 1). PCR amplification using total RNA isolated from TT-cells without prior reverse transcription was performed as described above. PCR without RT was unsuccessful in amplifying the products predicted from the cDNA sequence.

### Sensitivity of RT-PCR assay

RT-PCR assay sensitivity was determined using mixed aliquots of total RNA isolated from TT-cells and lymphocytes of a negative control patient. Assuming that one TT-cell contains 10 pg total RNA, we could detect the equivalent of 200 TT-cells/ml blood using calcitonin PCR and 50 TT-cells/ml blood using carcinoembryonic antigen PCR by agarose gel electrophoresis (Figure 2).

### Evaluation of RT-PCR assay in patients and controls

We evaluated 11 patients with medullary thyroid cancer, 7 of them with known metastasis (local recurrence), and 32 control patients without clinical signs or a history of thyroid disease. RT-PCR on all blood samples was performed using primers for calcitonin and carcinoembryonic antigen, respectively. Synthesis of cDNA was achieved in all cases, as determined by successful amplification of GAPDH cDNA in each sample.

Using primers for calcitonin, we detected calcitonin mRNA in peripheral blood of 0/11 patients with MTC and 0/32 controls. Using primers for CEA, we detected CEA-mRNA in peripheral blood of 8/11 (72.7%) patients with MTC and 11/32 (34.4%) controls ($P$ = 0.038; Fisher-exact-test; Table 2).

Figure 3 exemplarily shows a patient with sporadic medullary thyroid carcinoma and a nearly 10-year history of the disease. After multiple surgical interventions, tumour mass increased rapidly and the patient was no longer operable. Peripheral blood samples taken from this patient over a period of 6 months were always tested negative for calcitonin but positive for carcinoembryonic antigen mRNA. Calcitonin serum level was > 16 500 pg/ml (normal value < 20 pg/ml) and carcinoembryonic antigen serum level was 141 ng/ml (normal value < 4 ng/ml).

**Table 2** RT-PCR positivity rates for carcinoembryonic antigen mRNA in patients with MTC and controls. Significant correlation between existence of medullary thyroid carcinoma and a positive CEA RT-PCR result ($P$ = 0.038; Fisher-exact-test)

| | RT-PCR positive cases | | |
|---|---|---|---|
| | **No. of cases** | **No.** | **%** |
| Patients with MTC | 11 | 8 | 72.7 |
| Controls | 32 | 11 | 34.4 |

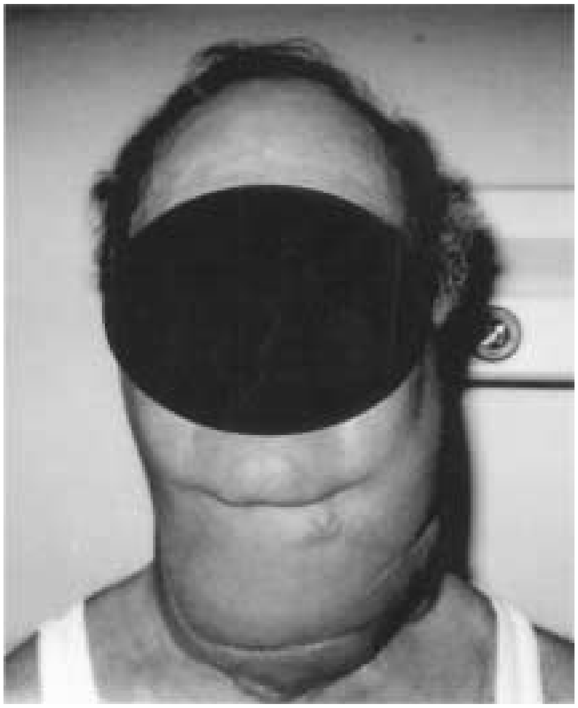

**Figure 3** Patient with sporadic medullary thyroid carcinoma and a nearly 10-year history of the disease. After multiple surgical interventions, tumour mass increased rapidly and the patient was no longer operable. Peripheral blood samples taken from this patient over a period of 6 months were always negative tested for calcitonin but positive for carcinoembryonic antigen mRNA. Calcitonin serum level was >16 500 pg/ml (normal value < 20 pg/ml) and carcinoembryonic antigen serum level was 141 ng/ml (normal value < 4 ng/ml)

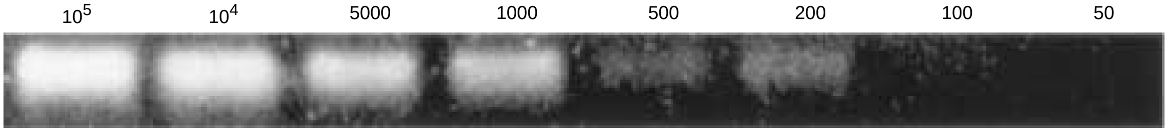

**Figure 2** Calcitonin RT-PCR sensitivity assay. Total RNA isolated from TT cell line was mixed with total RNA from peripheral-blood mononuclear cells. Sensitivity after 30 cycles of PCR amplification is shown. By ethidium bromide staining, calcitonin mRNA equivalent to 200 cells/ml blood was visualised

 *© 2001 Cancer Research Campaign*

### DISCUSSION

Diagnosis of local disease recurrence or metastasis in medullary thyroid carcinoma (MTC) remains to pose a diagnostic challenge in clinical practice. In this study, we evaluated whether detection of circulating calcitonin and CEA mRNA may add additional information to established studies for detection of recurrent disease. Molecular detection of tissue specific gene expression in peripheral blood is a new diagnostic approach and has been described as a tumour marker in different solid tumours (Johnson et al, 1995). In some malignant diseases – for example prostate cancer – detection of circulating tumour cells could be shown to precede the detection of secondary disease by serum markers determined by immunoassays (Ghossein et al, 1995). One study has been published, which has explored blood borne cytokeratin 20 mRNA as a potential tumour marker in thyroid cancer, especially MTC (Weber et al, 2000). To date, calcitonin and carcinoembryonic antigen mRNA have only been described as diagnostic tools for preoperative diagnosis of MTC in leftover cells from fine needle aspiration biopsy (Takano et al, 1999; Bugalho et al, 2000).

Using RT-PCR, calcitonin mRNA could neither be detected in peripheral blood of patients with MTC nor in controls in the present study, although serum levels of calcitonin (CT) were elevated in all patients with MTC and disease recurrence. Furthermore, multiple peripheral blood samples could be analysed for CT mRNA in a patient with known metastasis and massive tumour mass in the neck (Figure 3). Despite the fact that CT serum levels were highly increased in this patients, detection of circulating CT mRNA in this patient failed over a period of 6 months.

Although RT-PCR is a highly sensitive method of detect tumour and tissue-specific mRNA, for several reasons, detection of CT-mRNA in peripheral blood may have failed.

First, RT-PCR assay-sensitivity may be to low to detect circulating CT-mRNA. We could determine assay-sensitivity to detect 200 CT-mRNA experssing cells/ml whole blood. This sensitivity is in the range of other comparable studies (Ditkoff, et al 1996; Weber et al, 2000). From a technical point of view assy-sensitivity can be increased easily, but as we and others have described earlier, higher assay- sensitivities resulted in completely unspecific results (Tallini et al, 1998; Bojunga et al, 2000). Other investigators were able to detect cytokeratin 20 mRNA – suspected as a tumour specific marker – in peripheral blood of three out of eight patients with MTC and concluded that CK20 RT-PCR assay is able to detect circulating tumour cells in peripheral blood of thyroid carcinoma patients (Weber et al, 2000). However, the limitation of this study is that tissue specificity of the transcripts was not evaluated and as others have shown (Jung et al, 1999), the detection of micrometastasis by cytokeratin 20 RT-PCR is limited due to stable background transcription in granulocytes. In addition, blood samples from only eight patients with MTC were studied. In view of these results, further studies are necessary to determine whether cytokeratin 20 may be a candidate for detection of circulating tumour cells in MTC as well.

Second, medullary thyroid carcinoma exhibits several biological peculiarities. Before metastasis in a distant site can develop, tumour cells must circulate in the peripheral blood or lymphatic channels. MTC typically forms metastasis in the local lymph nodes of the neck region and of the proximal mediastinum (Bergholm et al, 1989). In patients with elevated basal calcitonin values during family screening programs, local lymph node involvement could be found in 50% of patients. Patients with clinically manifest tumours suffered from local metastases in 71% of cases (Wells et al, 1978). Local lymph node involvement is the strongest predictor for disease survival in MTC: in the study of Woolner et al (Woolner et al, 1969) the 10-year survival rate of patients without lymph node involvement was not significantly different to a reference population (85%), whereas survival was reduced to 42% in patients with lymph node involvement. So the reason for the failure of detecting CT-mRNA expressing cells in our patients with MTC may be that tumour cells may only circulate in peripheral blood very late in the natural history of the disease. Furthermore, occurrence of negative peripheral blood samples in patients with metastatic disease has been noted in other tumour models, and intermittent release of tumour cells in the bloodstream may be one additional explanation (Ghossein and Rosai, 1996).

Using primers specific for CEA, we could detect CEA mRNA significantly more often in patients with MTC (72,7%) than in controls (34,4%; $P < 0.05$). However, specificity and sensitivity are major concerns in RT-PCR systems for the detection of tumour cells in peripheral blood. Circulating CEA mRNA has widely been used in detection of metastasis in colo-rectal carcinoma and conflicting data have been published concerning false positive results for CEA mRNA in control patients: two groups using identical primer sequences did not find false positive cases in their studies and therefore reached assay specificity of 100% (Gerhard et al, 1994; Taniguchi et al, 2000), whereas other groups reported false positive rates between 23% in bone marrow samples from healthy controls (Jonas et al, 1996) and 26% in peripheral blood samples from healthy controls (Zippelius et al, 1997). Using our CEA RT-PCR assay with a sensitivity of 50 cells/ml blood, we were able to detect circulating CEA mRNA in 34% of healthy controls. The reason for this high percentage of RT-PCR positive controls remains unclear. One reason may be that non-specific low level expression of a variety of genes in different cell types, or illegitimate transcription, also occur in peripheral-blood leukocytes. Therefore, detection of CEA mRNA in peripheral blood might reflect detection of non-specific or illegitimate transcription in cell types of non-c-cell origin – e.g., lymphocytes or granulocytes – as it has been described earlier (Chelly et al, 1989). Multiple peripheral blood samples could be analysed for CEA mRNA from the patient mentioned above (Figure 3). CEA serum levels were also markedly increased in this patients and he was tested positive for circulating CEA mRNA several times over a period of 6 months.

Together, our results demonstrate that calcitonin mRNA does not seem to be a candidate for the detection of circulating tumour cells in MTC. However, in contrast to circulating calcitonin mRNA, our data suggest that the use of carcinoembryonic mRNA may provide a significant improvement in diagnosis of recurrent disease in MTC. Additional studies with quantitative assay format, larger groups of patients with long-term clinical follow up and establishment of cut-off values are necessary to further define the clinical value and diagnostic sensitivity and specificity of CEA mRNA RT-PCR assays in detecting disease recurrence in MTC.

### REFERENCES

Altschul SF, Gish W, Miller W, Myers EW and Lipman DJ (1990) Basic local alignment search tool. *J Mol Biol* **215**: 403–410

Bergholm U, Adami HO, Bergstrom R, Johansson H, Lundell G, Telenius-Berg M and Akerstrom G (1989) Clinical characteristics in sporadic and familial

© 2001 Cancer Research Campaign 

medullary thyroid carcinoma. A nationwide study of 249 patients in Sweden from 1959 through 1981. *Cancer* **63**: 1196–1204

Bojunga J, Röddiger S, Stanisch M, Kusterer K, Kurek R, Renneberg H, Adams S, Lindhorst E, Usadel K-H and Schumm-Draeger PM (2000) Molecular detection of thyroglobulin mRNA transcripts in peripheral blood of patients with thyroid disease by RT-PCR. *Br J Cancer* **82**: 1650–1655

Bracq S, Machairas M, Clement B, Pidoux E, Andreoletti M, Moukhtar MS and Jullienne A (1993) Calcitonin gene expression in normal human liver. *FEBS Lett* **331**: 15–18

Bugalho MJGM, Mendonca E and Sobrinho LG (2000) Medullary thyroid carcinoma: an accurate pre-operative diagnosis by reverse transcription-PCR. *Eur J Endocrinol* **143**: 335–338

Chelly J, Concordet JP, Kaplan JC and Kahn A (1989) Illegitimate transcription: transcription of any gene in any cell type. *Proc Natl Acad Sci USA* **86**: 2617–2621

Ditkoff BA, Marvin MR, Yemul S, Shi YJ, Chabot J, Feind C and Gerfo PL (1996) Detection of circulating thyroid cells in peripheral blood. *Surgery* **120**: 959–965

Gerhard M, Juhl H, Kalthoff H, Schreiber HW, Wagener C and Neumaier M (1994) Specific detection of carcinoembryonic antigen-expressing tumor cells in bone marrow aspirates by polymerase chain reaction. *J Clin Oncol* **12**: 725–729

Ghossein RA, Scher HI, Gerald WL, Kelly WK, Curley T, Amsterdam A, Zhang ZF and Rosai J (1995) Detection of circulating tumor cells in patients with localized and metastatic prostatic carcinoma: clinical implication. *J Clin Oncol* **13**: 1195–1200

Ghossein RA and Rosai J (1996) Polymerase chain reaction in the detection of micrometastases and circulating tumor cells. *Cancer* **78**: 10–16

Hundahl SA, Fleming ID, Fremgen AM and Menck HR (1998) A National Cancer Data Base report on 53,856 cases of thyroid carcinoma treated in the U.S., 1985–1995. *Cancer* **83**: 2638–2648

Johnson PW, Burchill SA and Selby PJ (1995) The molecular detection of circulating tumour cells. *Br J Cancer* **72**: 268–276

Jonas S, Windeatt S, Boateng A, Fordy C and Allen-Mersh TG (1996) Identification of carcinoembryonic antigen-producing cells circulating in the blood of patients with colorectal carcinoma by reverse transcriptase polymerase chain reaction. *Gut* **39**: 717–721

Jung R, Petersen K, Kruger W, Wolf M, Wagener C, Zander A and Neumaier M (1999) Detection of micrometastasis by cytokeratin 20 RT-PCR is limited due to stable background transcription in granulocytes. *Br J Cancer* **81**: 870–873

Marsh DJ, Learoyd DL and Robinson BG (1995) Medullary thyroid carcinoma: recent advances and management update. *Thyroid* **5**: 407–424

Takano T, Miyauchi A, Matsuzuka F, Liu G, Higashiyama T, Yokozawa T, Kuma K and Amino N (1999) Preoperative diagnosis of medullary thyroid carcinoma by RT-PCR using RNA extracted from leftover cells within a needle used for fine needle aspiration biopsy. *J Clin Endocrinol Metab* **84**: 951–955

Tallini G, Ghossein RA, Emanuel J, Gill J, Kinder B, Dimich AB, Costa J, Robbins R, Burrow GN and Rosai J (1998) Detection of thyroglobulin, thyroid peroxidase, and RET/PTC1 mRNA transcripts in the peripheral blood of patients with thyroid disease. *J Clin Oncol* **16**: 1158–1166

Taniguchi T, Makino M, Suzuki K and Kaibara N (2000) Prognostic significance of reverse transcriptase-polymerase chain reaction measurement of carcinoembryonic antigen mRNA levels in tumor drainage blood and peripheral blood of patients with colorectal carcinoma. *Cancer* **89**: 970–976

Weber T, Lacroix J, Weitz J, Amnan K, Magener A, Holting T, Klar E, Herfarth C and von Knebel D (2000) Expression of cytokeratin 20 in thyroid carcinomas and peripheral blood detected by reverse transcription polymerase chain reaction. *Br J Cancer* **82**: 157–160

Wells SAJ, Baylin SB, Gann DS, Farrell RE, Dilley WG, Preissig SH, Linehan WM and Cooper CW (1978) Medullary thyroid carcinoma: relationship of method of diagnosis to pathologic staging. *Ann Surg* **188**: 377–383

Woolner LB, Beahrs OH, Black MB, McCanahey WM and Keating FR (1969) Long-term survival rates. *UICC Monogr Ser* **12**: 326–330

Zippelius A, Kufer P, Honold G, Kollermann MW, Oberneder R, Schlimok G, Riethmuller G and Pantel K (1997) Limitations of reverse-transcriptase polymerase chain reaction analyses for detection of micrometastatic epithelial cancer cells in bone marrow. *J Clin Oncol* **15**: 2701–2708

*© 2001 Cancer Research Campaign*
